# Functional capacity and rehabilitation strategies in Covid-19 patients: current knowledge and challenges

**DOI:** 10.1590/0037-8682-0789-2020

**Published:** 2021-01-29

**Authors:** Aline Xavier Frota, Marcelo Carvalho Vieira, Carla Cristiane Santos Soares, Paula Simplício da Silva, Gilberto Marcelo Sperandio da Silva, Fernanda de Souza Nogueira Sardinha Mendes, Flavia Mazzoli-Rocha, Henrique Horta Veloso, Ananda Dutra da Costa, Cristiane da Cruz Lamas, Claudia Maria Valete-Rosalino, Tatiana Rehder Gonçalves, Henrique Silveira Costa, Luiz Fernando Rodrigues, Mauro Felippe Felix Mediano

**Affiliations:** 1 Fundação Oswaldo Cruz, Instituto Nacional de Infectologia Evandro Chagas, Rio de Janeiro, RJ, Brasil.; 2 Instituto Nacional de Cardiologia, Departamento de Pesquisa e Educação, Rio de Janeiro, RJ, Brasil.; 3 Universidade Federal do Rio de Janeiro, Departamento de Otorrinolaringologia e Oftalmologia, Rio de Janeiro, RJ, Brasil.; 4 Conselho Nacional de Desenvolvimento Científico e Tecnológico, Programa de Produtividade em Pesquisa, Brasília, DF, Brasil.; 5 Universidade Federal do Rio de Janeiro, Instituto de Medicina Social, Rio de Janeiro, RJ, Brasil.; 6 Universidade Federal dos Vales do Jequitinhonha e Mucuri, Departamento de Fisioterapia, Diamantina, MG, Brasil.; 7 Universidade Federal do Estado do Rio de Janeiro, Departamento de Ciências Fisiológicas, Rio de Janeiro, RJ, Brasil.

**Keywords:** Coronavirus infection, Covid-19, Exercise therapy, Complications, Comprehensive care, Rehabilitation

## Abstract

Covid-19 is a novel infectious disease whose spectrum of presentation ranges from absence of symptoms to widespread interstitial pneumonia associated with severe acute respiratory syndrome (SARS), leading to significant mortality. Given the systemic pattern of Covid-19, there are many factors that can influence patient's functional capacity after acute infection and the identification of such factors can contribute to the development of specific rehabilitation strategies. Pulmonary impairment is the primary cause of hospitalization due to Covid-19, and can progress to SARS as well as increase length of hospitalization. Moreover, cardiac involvement is observed in approximately 30% of hospitalized patients, with an increased risk of acute myocarditis, myocardial injury, and heart failure, which may compromise functional capacity in the long-term. Thromboembolic complications have also been reported in some patients with Covid-19 and are associated with a poor prognosis. Musculoskeletal complications may result from long periods of hospitalization and immobility, and can include fatigue, muscle weakness and polyneuropathy. Studies that address the functional capacity of patients after Covid-19 infection are still scarce. However, based on knowledge from the multiple systemic complications associated with Covid-19, it is reasonable to suggest that most patients, especially those who underwent prolonged hospitalization, will need a multiprofessional rehabilitation program. Further studies are needed to evaluate the functional impact and the rehabilitation strategies for patients affected by Covid-19.

## INTRODUCTION

The betacoronavirus SARS-CoV-2, discovered in 2019 in China, is one of the six known coronavirus species capable of infecting humans^1^ ^-^ ^3^. Of these, four are associated with the development of mild flu syndrome, with typical symptoms of a common cold^1^. However, SARS-CoV-2, which is highly transmissible, mainly through respiratory droplets, aerosols and direct contact with contaminated objects, is the etiological agent of Covid-19 (coronavirus disease 2019), a disease that has no specific treatment and whose spectrum of presentation varies from absence of symptoms to widespread interstitial pneumonia associated with severe acute respiratory syndrome (SARS), resulting in 5% to 10% mortality^2^ ^-^ ^6^. 

In Brazil, there were about 5 million cases of Covid-19 confirmed by November 2020^7^. Most infected individuals remain asymptomatic or have a mild or moderate form of the disease (85%), with non-specific symptoms such as fever, cough, myalgia, sputum and fatigue^4^ ^,^ ^8^ ^,^ ^9^. However, about 15% of individuals with Covid-19 may develop the severe form of the disease that requires hospitalization and ventilatory support, with significant lung damage and progressive hypoxemic respiratory failure, characteristics of SARS. These cases represent the main cause of hospitalizations and death^4^ ^,^ ^8^ ^-^ ^10^. The mortality rate due to Covid-19 in Brazil is 76.4 deaths per 100,000 inhabitants^7^.

**FIGURE 1:**
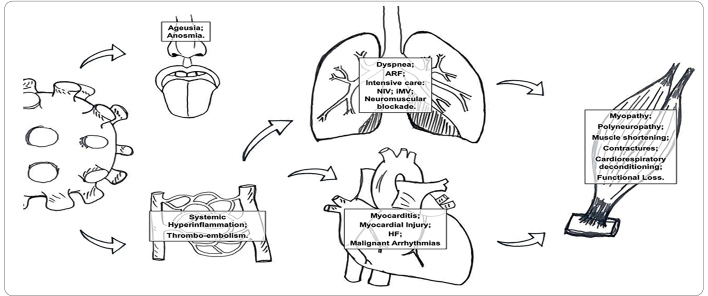
Main complications from COVID-19 infection that impact functional capacity.

Numerous factors have been attributed to increased disease severity; among them are the prothrombotic potential and the development of systemic hyperinflammation, known as a cytokine storm, which leads to rapid failure of multiple organs (liver, kidneys, lungs and heart) and is associated with the clinical symptoms of hypoxia, such as dyspnea, fatigue and shortness of breath^9^ ^,^ ^11^ ^-^ ^13^. The main complications resulting from Covid-19 infection are described in Figure 1.

Among patients who develop the disease in its most severe form, 15% require hospitalization and 5% require further advanced life support in intensive care units (ICU) over prolonged periods^14^. Although the negative impact of prolonged hospitalization on post-discharge functional capacity is widely recognized, the challenge for the Covid-19 survivors may be even greater, as both the pathophysiology and the necessity for prolonged treatment during the acute severe stage of the disease can cause secondary organ damage that compromises functional recovery and the capacity to perform activities of daily living (ADL)^9^ ^,^ ^15^. Considering that Covid-19 can affects different physiological systems, with more than 80% of the survivors presenting some long-term functional limitation months after initial symptom onset, several factors can influence the patient's functional capacity after the acute infection and the identification of such factors can help the development of specific rehabilitation strategies for these patients^6^ ^,^ ^12^ ^,^ ^13^ ^,^ ^16^. 

## FACTORS THAT INFLUENCE FUNCTIONAL CAPACITY AFTER COVID-19

### Pulmonary complications

Pulmonary complications of Covid-19 are similar to the previous epidemics caused by other identified coronaviruses - namely the Severe Acute Respiratory Syndrome (SARS-CoV) and Middle East Respiratory Syndrome (MERS-CoV)^6^. SARS is characterized by an acute inflammatory response with cytokine release that results in alveolar and capillary endothelial damage with consequences that include non-cardiogenic pulmonary edema^17^. Biopsy samples from lung tissues of Covid-19 patients identified viral particles in the cytoplasm of type II pneumocytes and demonstrated bilateral diffuse alveolar lesions with exudates and cellular fibromyxoid features^1^ ^,^ ^18^. 

Prolonged mechanical ventilation is necessary for patients that develop severe disease symptoms, but can cause secondary lung injuries in some cases, leading to complications such as edema, pulmonary inflammation and abnormal surfactant function, decreasing lung compliance and reducing gas exchange^19^. In the recovery phase, patients may experience residual lung damage, such as parenchymal consolidation, in which the alveolar walls develop fibrosis due to parenchymal repair processes, directly affecting respiratory function^1^ ^,^ ^19^ ^,^ ^20^.

In terms of lung function, no reductions in spirometry values have been observed after Covid-19 infection, although significant changes were observed in diffusion capacity and lung volume^21^. However, about 43% of patients may develop obstructive pulmonary patterns and about 53% develop restrictive pulmonary patterns within the first year after hospitalization; this can negatively influence functional capacity and quality of life^15^ ^,^ ^22^. Long-term follow-up to monitor lung function is advised for patients with persistent respiratory symptoms to provide functional parameters on which pulmonary rehabilitation can be based^23^.

### Cardiac complications

Damage to the cardiovascular system inflicted by Covid-19 is likely to be multifactorial and can result from an imbalance between high metabolic demand and low cardiac reserve, systemic inflammation and thrombogenesis, or direct cardiac injury caused by invasion of the virus into the myocardium^14^ ^,^ ^24^ ^,^ ^25^. High concentrations of cytokines and inflammatory markers associated with myocardial interstitial macrophage infiltration may promote myocarditis with myocardial injury, heart failure, cardiogenic shock, and malignant arrhythmias, that may persist after the acute phase^14^ ^,^ ^25^ ^-^ ^28^. Usually, acute cardiac injury is more severe in individuals with previous comorbidities, such as hypertension, diabetes mellitus, and cardiovascular disease, as well as those who require ventilatory support during the acute infection phase. These factors are also associated with a worse prognosis^22^. 

Cardiac injury was observed in up to 30% of hospitalized patients affected by Covid-19, and was associated with a higher rate of mortality^29^ ^,^ ^30^. Moreover, the cardiac complications related to Covid-19, together with the prolonged immobility period during hospitalization, are associated with a marked decrease in cardiorespiratory capacity, which may reduce the ability to perform ADL and increase the risk of cardiac events after discharge^12^ ^,^ ^15^ ^,^ ^20^. The deleterious impact of immobility on cardiorespiratory fitness was assessed in the classic Dallas Bed Rest study, showing a 30% reduction in maximum cardiorespiratory capacity after 3 weeks of bed rest^31^.

### Thromboembolic complications

An important marker of poor prognosis in Covid-19 is the pro-thrombotic state^32^. A meta-analysis previously demonstrated that the incidence of thromboembolic events in patients with Covid-19 is around 22%^33^. Patients may present with hypercoagulability, placing them at increased risk of thromboembolic events such as pulmonary thromboembolism and stroke^15^ ^,^ ^32^ ^,^ ^34^ ^,^ ^35^. A significant increase in D-dimer, an important marker of deep venous thrombosis, is also possible, and may be related to the development of intravascular hypercoagulability (71%)^36^ ^,^ ^37^. The associations between immobility, systemic inflammation, platelet activation, endothelial dysfunction, and blood stasis can lead to abnormalities in coagulation^38^.

Covid-19 patients presenting with the most severe form of the disease have also been known to develop disseminated intravascular coagulation (DIC) and are at an increased risk of death^36^. DIC occurs as consequence of an acute inflammatory response or sepsis, which leads to endothelial and tissue damage and, ultimately, to multiple organ failure^36^ ^,^ ^37^. Thus, the identification of patients with coagulopathy is of paramount importance to determine if anticoagulant therapy should be started to improve prognosis^33^. 

In a retrospective study, Tang et al. (2020) evaluated the coagulation profiles and outcomes of patients with severe Covid-19 and observed that treatment with heparin was associated with lower mortality rates in cases where the dosage of D-dimer was greater than 3 µg/mL^39^. In addition, Paranjpe et al. (2020) found that treatment with anticoagulants was associated with increased survival of patients on mechanical ventilation^40^.

### Peripheral musculoskeletal disorders

Little is known about long-term musculoskeletal disorders associated with Covid-19. However, based on knowledge from previous studies investigating patients who underwent prolonged hospitalization, patients with Covid-19 may experience chronic fatigue and musculoskeletal disorders after hospital discharge, representing an important public and occupational health problem that requires specific rehabilitation care^22^ ^,^ ^41^.

Fatigue is a common symptom of the acute phase of Covid-19 that may persist for several months after hospital discharge in up to 10 to 20% of patients^42^. Similar to other postviral conditions, fatigue in Covid-19 may be associated with persistent high levels of IL-6 and IL-10 in the chronic phase, as a result of the cytokine storm during the acute phase^43^. 

Musculoskeletal disorders and reduced muscle strength have been observed in critically ill patients with prolonged hospitalization due to three main possibilities: 1) muscle hypoxia, in which the inadequate systemic and peripheral muscle perfusion caused by the disease can increase anaerobiosis, raising lactate levels and impairing muscle function; 2) prolonged immobility, common in patients admitted to ICU, which is associated with marked reductions in muscle strength due to loss of functional units (sarcomeres) from lack of active movement, which in turn leads to postural instability, muscle shortening and contractures^9^ ^,^ ^15^ ^,^ ^41^ ^,^ ^44^; 3) the use of steroids and neuromuscular blocking agents over a long-term period, which can lead to post-hospitalization polyneuropathy and myopathy^41^ ^,^ ^44^.

In the case of Covid-19, peculiarities in the treatment of SARS, such as the need for intense neuromuscular blocking to perform positive end-expiratory pressure (PEEP) titration, and thereby increase alveolar recruitment, in addition to prone positioning can lead to a more prolonged use of neuromuscular blockers that will further increase sarcopenia (loss of muscle mass), a condition that is already typical in patients hospitalized in ICU^9^ ^,^ ^41^. Moreover, the use of corticosteroids is indicated in patients with moderate to severe Covid-19 due to the severity of lung injury, further increasing muscle wasting and weakness^45^ ^,^ ^46^. Therefore, Covid-19 patients can develop a state of physical and functional impairment that can last over a long-term period beyond discharge, negatively affecting their quality of life and increasing the risk of mental disorders such as anxiety and depression^44^ ^,^ ^47^.

### Olfactory and gustatory abnormalities

Olfactory dysfunction (OD) and gustatory dysfunction (GD) have been identified as initial and frequent symptoms in Covid-19^48^ ^,^ ^49^. Therefore, the presence of ageusia (loss of taste) and/or anosmia (loss of smell) should be considered in Covid-19 diagnosis^49^. Ageusia and anosmia are associated with each other and are reported in 30% to 80% of patients with Covid-19. In 12% of cases, OD is the first symptom^49^ ^-^ ^52^. Furthermore, anosmia has been associated with the mild to moderate form of Covid-19^50^ ^,^ ^53^. Serious OD and GD have been reported to affect 60% and 40% of patients, respectively^52^. The duration of anosmia and hyposmia is usually at least five days, with the most significant improvements in taste function occurring during the first 10 days and in olfactory function between 10 and 20 days after the onset of symptoms. The majority of patients recover completely within 30 days^48^ ^,^ ^51^ ^,^ ^53^. However, one-third of patients have only partial improvement in the severity of OD/GD, and in around 5% of patients, the symptoms remain unchanged or still severe after 60 days of disease onset^52^ ^,^ ^53^.

## REHABILITATION STRATEGIES

Rehabilitation is a multidisciplinary intervention that aims to minimize disabilities, recover functional independence, and improve the ability to perform ADL^9^. Specifically, in Covid-19, the aims are to improve functional capacity, increase quality of life, facilitate the social reintegration after hospitalization, decrease fatigue, dyspnea, ageusia, anosmia and anorexia, and improve the ability to perform ADL^47^ ^,^ ^54^. Therefore, early rehabilitation activities should be offered to patients after discharge in order to minimize the most deleterious consequences of Covid-19^55^ ^,^ ^56^. 

Literature regarding rehabilitation after Covid-19 remains scarce^57^. The rehabilitation process must take into account the peculiarities of the disease to meet individual needs. Every rehabilitation program must consider the comorbidities that can affect the clinical progression or the ability to perform ADL^22^. In this context, a multidisciplinary approach including a comprehensive evaluation and thorough training is of paramount importance for optimal results^47^ ^,^ ^54^. Rehabilitation programs can be carried out at home, in an outpatient clinic or even in a remotely supervised environment, as already performed in some countries^22^ ^,^ ^58^.

### Respiratory rehabilitation

Respiratory rehabilitation aims to improve quality of life by managing dyspnea, improving exercise tolerance and increasing functional capacity^20^. After initial recovery from Covid-19, especially for those who required hospitalization in ICU, it is possible that some patients may experience respiratory muscle dysfunction, as well as pulmonary restriction or obstruction to varying extents, affecting peripheral muscle function and respiratory conditioning^15^ ^,^ ^22^. 

The respiratory rehabilitation process consists of diaphragmatic exercises, forced expiration and cough exercises, and respiratory muscle training with linear load devices, in addition to accessory muscle stretching and physical training^9^ ^,^ ^20^ ^,^ ^58^ ^,^ ^59^. The duration of a respiratory rehabilitation program will depend on the patient`s clinical condition and comorbidities, though some studies support timeframes of at least 6 to 8 weeks to maximize benefits^20^ ^,^ ^22^ ^,^ ^59^. In a randomized clinical trial, Liu et al. (2020) demonstrated significant improvements in lung function and quality of life after a 6-week respiratory rehabilitation program in a sample of elderly patients recovering from Covid-19^20^.

Submaximal tests (e.g. 6-minute walk test) are useful to assess patient`s functional capacity and to guide the prescription of exercise. The 6-minute walk test (6MWT) is simple, easy to perform, inexpensive and widely used in clinical routine^60^. However, some patients, especially those in need of higher fractions of inspired oxygen (FIO2) (≥40%), were unable to perform the 6MWT due to dyspnea and shortness of breath, even for minimal activities. In an Italian rehabilitation unit^61^, only 44% of the patients were able to walk, all with dyspnea grades 4 and 5 on the Modified Medical Research Council dyspnea scale and with great disability, as determined by the Barthel index. In these cases, a potentially useful tool is the 1-min sit to stand test (1-min STS), which assesses, in addition to functional capacity, the muscular strength of the lower limbs, balance and risk of fall, with the advantage of requiring little space and less time^61^ ^-^ ^62^. The 1-min STS test proved to be a valid alternative to 6-minute walk test in patients who have undergone lung transplantation^63^. In the setting of the Covid-19 pandemic, performance on the 1-min STS test at hospital admission and at home discharge was reported in one previous study^64^, together with the Short Physical Performance Battery (to assess lower extremity function) and Barthel index measurement (to assess performance in ADL). Finally, the 1-min STS test has also been used in telerehabilitation programs, as it is simple and facilitates the follow-up of patients after discharge^62^.

Additionally, Curci et al. (2020) suggested that, in critically ill patients, functional capacity and disability can be evaluated by the Chelsea Critical Care Physical Assessment Tool (CPAx)^65^. The CPAx was developed by Corner et al. (2013) to evaluate the functionality of patients admitted to the ICU^66^. The instrument is composed of 10 items related to physical function and is graded with six ratings ranging from complete dependence to independence and a total score ranging from 0 to 50, with a score of 50 representing complete independence. The CPAx evaluates the respiratory function, cough ability, bed mobility, ability to change position, standing balance, sitting, stepping, and handgrip strength. Although it has not been used in patients recovering from Covid-19, the CPAx assesses a wide spectrum of functional impairments potentially caused by the disease, and therefore may be an useful tool for monitoring patients^66^.

### Cardiovascular rehabilitation

It is possible that some patients will require cardiovascular rehabilitation after Covid-19 infection due to cardiovascular consequences associated with the disease. Cardiac rehabilitation aims to improve functional capacity and quality of life, as well as to reduce morbidity and mortality among those with associated comorbidities and heart disease^22^ ^,^ ^29^ ^,^ ^67^. 

Cardiac impairments should be considered in patients after Covid-19 regardless of the severity of hospitalization. Complementary tests such as electrocardiogram, echocardiogram, and exercise test (with or without gas exchange analysis) to assess the cardiac and cardiopulmonary function are advised before starting a rehabilitation program^22^ ^,^ ^68^.

In the presence of concomitant heart disease, specific cardiovascular rehabilitation programs are indicated^22^. According to the current Brazilian Cardiovascular Rehabilitation Guideline (2020), a cardiovascular rehabilitation program should include aerobic, resistance (for peripheral and respiratory muscles), stretching, and balance exercises that are prescribed according to the individual functional capacity and clinical condition^68^. The guideline recommends 150 minutes per week of moderate-to-vigorous exercise, distributed across 3 to 5 sessions, which should be individually prescribed according to clinical and functional parameters. In terms of intensity, it is recommended 70 to 85% of the peak heart rate obtained during the initial exercise test. During training sessions, it is important to monitor all vital signs (heart rate, blood pressure, and oxygen saturation) routinely before, during, and after exercise to detect and prevent potential adverse events^68^. The Borg effort perception scale may be used to control exercise intensity, beginning with low intensity at the start of training program (< 3 METs, a metabolic equivalent of task that is the ratio at which a person expends energy related to resting metabolism) and gradually increasing based on patient`s tolerance^22^ ^,^ ^68^.

Inspiratory muscle training (IMT) can be added to conventional cardiovascular rehabilitation to optimize inspiratory muscle strengthening, improving functional capacity and quality of life, as already described for patients with cardiovascular disorders from other etiologies^20^ ^,^ ^69^ ^,^ ^70^ ^,^ ^71^.

### Peripheral musculoskeletal rehabilitation

Individuals who underwent prolonged hospitalization due to Covid-19 infection may develop long-term motor disability, with secondary effects such as peripheral muscle weakness, fatigue and atrophy^41^.

In this setting, the Chinese Association of Rehabilitation Medicine (2020) published a document with recommendations for functional rehabilitation of patients with Covid-19 indicating aerobic, resistance and functional exercises to optimize physical functioning^59^. The document recommends aerobic exercises 3 to 5 days a week for 20 to 30 minutes according to the patient's tolerance, suggesting intermittent exercises for those patients that present fatigue at minimum efforts. Progressive resistance training 2 to 3 times a week is recommended for peripheral muscles, with 8 to 12 maximum repetitions and interval of 2 minutes between one to three sets. A 5 to 10% weekly gain in strength is expected. It is also recommended to include instrumental training to minimize the obstacles encountered to perform ADL^59^.

### Speech therapy rehabilitation

Patients admitted to intensive care due to Covid-19 who underwent prolonged invasive mechanical ventilation and tracheostomy are at high risk of oropharyngeal dysphagia. Post-extubation swallowing sequelae are frequently observed, such as glottic edema, tracheal lumen stenosis, and laryngeal muscle sensitivity^48^ ^,^ ^72^ ^,^ ^73^

Speech therapy is responsible for the rehabilitation of patients with dysphagia, as well as speech disorders and some respiratory disorders. Some techniques can be applied to rehabilitate patients after Covid-19; these include orofacial exercises and exercises to facilitate glottic coaptation, improve laryngeal excursion, assist pneumophonoarticulatory coordination, increase maximum phonation time, and postural maneuvers to facilitate swallowing^73^.

### Olfactory and gustatory rehabilitation

In case of absent or minimal OD, GD, or both, the patient typically does not require assistance. However, when the recovery of symptoms after the disease is only partial, the patient should be referred for otorhinolaryngological evaluation and nasal endoscopy to rule out another potential nasosinusal disease such as chronic rhinosinusitis with or without nasal polyps. More specific tools like the SNOT-22 and chemosensory complaint score can also be used. When symptoms remain unresolved and the patient presents with severe OD, GD, or both, other tests may be necessary; these may include allergy testing, previous active rhinomanometry, nasal cytology, discriminatory odor and threshold tests, and electrogustometry, in addition to a brain magnetic resonance imaging exam^52^ ^,^ ^53^. To avoid long-term morbidity, specific therapies should be initiated in patients with moderate to severe olfactory disorders beginning 20 days after the onset of the disease^53^, such as intranasal steroids and olfactory training. In severe cases, measures like installing gas and smoke detectors at home and psychological counseling may be necessary^51^.

### Nutritional orientation

During infection with Covid-19, it is important to attend to the patient's nutritional status, as this directly affects health status and recovery. Tailored nutritional therapy is important for clinical improvement together with other medical and multiprofessional therapies^59^. Moreover, social isolation due to the Covid-19 pandemic may promote changes in daily routines that influence emotional disturbances, such as stress and anxiety, leading to changes in eating behaviors, alcohol intake, and increasing the consumption of comfort or ultra-processed foods, which are rich in hydrogenated oils, salt, sugar, other fats, and chemical additives^74^ ^-^ ^76^. These aforementioned changes can ultimately lead to metabolic and hemodynamic dysregulations (blood glucose, lipids, and blood pressure) and weight gain. In addition, the pandemic has also affected several economic sectors, decreasing or suppressing sources of income and subsequently increasing the risk of food insecurity^76^.

Psychological, economic and social issues must also be considered when monitoring patients during the rehabilitation phase after Covid-19^77^. The gradual return to activity should be followed by assessments of nutritional status, eating behavior, biochemical and metabolic parameters, food security, and nutritional support to optimize clinical management. Nutritional orientation provided during periods of social isolation should be provided to promote hygiene, as well as optimal food safety practices and healthy food choices. Nutritional orientation should provide education on healthy eating in consideration of the socioeconomic conditions of each patient, weight maintenance needs, and control of comorbidities, thus stimulating a healthy lifestyle.

### Pharmaceutical care

Considering that many of the patients can be under polypharmacy after Covid-19 infection, pharmaceutical counseling on appropriate drug usage and patient education may be beneficial^78^. Special attention must be given to adverse drug reactions from the use of some drugs such as anticoagulants, nonsteroidal anti-inflammatory, angiotensin-converting-enzyme inhibitors, and angiotensin II receptor blockers^79^ ^,^ ^80^. Close follow-up after hospital discharge is required for patients at higher risk of thromboembolism who may require anticoagulation therapy over a long period^23^. In this setting, pharmaceutical care can help patients to avoid inappropriate self-medication and improve the adherence to their well-prescribed medications.

## CONCLUSIONS

There are still few studies that address the functional capacity of patients after Covid-19 infection. However, based on the characteristics and mechanisms of the disease, and considering the multiple complications that can be encountered, it is possible to infer that most patients, especially those who have undergone prolonged hospitalization, will benefit from a multiprofessional rehabilitation program to recover their functional capacity and quality of life. However, more studies are needed to assess functional disability and its repercussions in patients affected by Covid-19.
